# Training integration in science, policy and practice: insights from designing and implementing integrative teaching and learning

**DOI:** 10.1057/s41599-026-06523-6

**Published:** 2026-01-29

**Authors:** Sabine Hoffmann, Bianca Vienni-Baptista

**Affiliations:** 1 Swiss Federal Institute of Aquatic Science and Technology, Dübendorf, Switzerland; 2 TdLab, ETH Zurich, Zurich, Switzerland

**Keywords:** Anthropology, Cultural and media studies, Science, technology and society

## Abstract

Inter- and transdisciplinary (ITD) research is increasingly valued for its contribution to solving complex problems by crossing the boundaries not only of different scientific disciplines, but also those of science, policy and practice. Integration across such boundaries is widely seen as the core challenge of ITD research and critical to the success or failure of ITD projects or programs. However, there are few studies that provide insights into *how to proactively lead* and *actively engage* in integration in ITD research, teaching and learning. There are even fewer studies that provide insights into *how to* *effectively train integration* in ITD higher education. Therefore, this article addresses two questions: *(i) How can we equip students, i.e. potential future leaders and team members of ITD projects or programs, with the key competencies necessary to lead and engage in integration across the boundaries of science, policy and practice? (ii) What lessons learned can we derive from training integration in ITD higher education in order to support study directors and lecturers aiming at equipping students with the necessary competencies in integration?* To answer these questions, we combine our conceptual insights from studying integration with empirical insights from leading and engaging in integration in ITD projects or programs. We derive principles for designing and implementing integrative teaching and learning and then exemplify how we apply such principles in our MSc course “Integration in Science, Policy and Practice: Inter- and Transdisciplinary Concepts, Methods, Tools” at ETH Zurich in Switzerland. We define method- and subject-specific competencies as well as social and personal competencies in integration, operationalize such competencies through competence-oriented learning objectives, outline teaching and learning activities, discuss learning outcomes and draw first lessons learned. We conclude by outlining ways forward that can support current and future study directors and lecturers in their efforts to design and implement integrative teaching and learning in ITD higher education.

## Introduction

Inter- and transdisciplinary (ITD) research is increasingly valued for its contribution to solving complex environmental and societal problems (Leitao, 2023) by crossing the boundaries not only of different scientific disciplines *(interdisciplinary research)*, but also those of science, policy and practice *(transdisciplinary research)* (Nowotny et al., 2001). Integration across such boundaries is often considered the “crux” of ITD research (Klein, 2008; Bruun et al., 2005) and critical to the success or failure of ITD research projects or programs (Defila et al., 2006; O’Rourke et al., 2016; Lux et al., 2019; Hoffmann et al., 2022a). It can be defined as the process of weaving different and previously unrelated perspectives together into a whole with a view to: a) enhancing scientific understanding of complex problems, b) generating policy- or practice-oriented solutions, and/or c) producing synthesis products tailored to the specific needs of different target audiences (Hoffmann et al., 2022b). “*Forming of* or *combining into*” a ‘whole’ seems incompatible with ITD research, where – as noted by O’Rourke et al. (2016, p. 67) – “integration often produces partial, intermediate combinations that no one would take to be a whole, especially in early stages”. That particular interpretation of a ‘whole’, “understood as the final, complete product” is not imposed on us here; rather, we emphasize the process of recognizing and establishing critical connections as well as acknowledging and – where possible – overcoming potential incompatibilities between perspectives (O’Rourke et al. (2016, p. 67).

This combination process that is at the heart of inter- and transdisciplinary integration (O’Rourke, 2025) involves iteration, reflection and continuous adaptation among leaders and team members of ITD projects or programs (National Academies of Sciences, 2018; Hoffmann et al., 2022b). It requires that both leaders and team members recognise, engage in, and build on the diversity of perspectives (Hoffmann et al., 2022b) and that they combine the different perspectives, understood as inputs, into “emergent and more comprehensive” outputs (O’Rourke, 2025). The integrated outputs that emerge from this iterative and reflexive process can include, but are not limited to, scientific publications, policy briefs, action guides, factsheets, videos, cartoons and/or new frameworks, concepts, methods or tools (Deutsch et al., 2025a; Hoffmann, 2024; Briers and Vienni-Baptista, 2024).

Acknowledging the importance of integration for ITD research, recent studies have emphasised that integration does not happen automatically, but needs to be *proactively led* and *actively engaged in* from the very beginning to ensure that research projects or programs live up to their ITD ambition and realise their integrative potential (Deutsch et al., 2025a; Berger, 2019; Defila et al., 2006; Gray, 2008; Lyall et al., 2011; Caviglia-Harris et al., 2021; Hoffmann et al., 2022b; Vienni-Baptista et al., 2020; Vienni-Baptista et al., 2024a). If not, the research process may evolve into parallel disciplinary efforts without integration of perspectives from science, policy and practice (usually referred to as multidisciplinary research) (Tress et al., 2005; O’Rourke et al., 2016) and thus without an integrated output at the end – or at most a multidisciplinary compilation of separate contributions from different disciplines, fields or sectors (Bruun et al., 2005).

Hence, “integration cannot be assumed to ‘just’ take place” (Hölscher et al., 2021, p. 14). This insight notwithstanding, few studies investigate integration in ITD projects or programs with a view to gaining conceptual and empirical insights into *how to proactively lead* and *actively engage* in integration (Deutsch et al., 2025b; Deutsch et al., 2025a). Following Boix Mansilla et al. (2015) and Pohl et al. (2021), these studies highlight that leading integration involves a range of challenges (Deutsch et al., 2025b; Deutsch et al., 2025a; Hoffmann, 2023): the *cognitive challenge* of understanding the distinct concepts, methods and tools from different disciplines, fields and sectors, recognizing their potentials and limitations, identifying synergies, and generating new knowledge by establishing critical connections between them (Jahn et al., 2012; Specht et al., 2015); the *social challenge* of acknowledging and – where possible – reconciling the different and sometimes competing interests, expectations and needs of the team members, (sub)projects and organisations involved (Jahn et al., 2012; Hoffmann et al., 2017a), and managing ‘essential tensions’ (Hackett, 2005), power dynamics and interpersonal conflicts (Oliver et al., 2019); the *emotional challenge* of building a climate of trust, respect, recognition and appreciation among team members (Cronin et al., 2011; Boix Mansilla et al., 2015) and providing a safe space that enables team members to expose oneself to the otherness of others and jointly engage in mutual learning (Freeth and Caniglia, 2019; Vilsmaier et al., 2015; Regeer et al., 2024).

On top of these cognitive, social and emotional challenges, recent studies indicate that leading integration implies the challenge of navigating positionality (Freeth and Vilsmaier, 2020) and balancing one’s own *supportive* (e.g., providing psychological, social and material support for creativity) and *creative contributions* to integration (e.g., generating, refining and linking creative ideas, including one’s own with those of others) (Deutsch et al., 2025a; Deutsch et al., 2021). Building on Mainemelis et al. (2015)’s definition of integrative leadership as an interplay of supportive and creative contributions from both leaders and team members (Deutsch et al., 2025a), leading integration in ITD projects or programs thus poses not only the challenge of navigating positionality and balancing one’s own creative and supportive contributions to integration, but also – and perhaps more importantly – the challenge of ensuring the creative and supportive contributions of team members in order that – in a joint collaborative effort of both leaders and team members – the integrative potential of ITD projects or programs are realized (Hoffmann et al., 2024). In other words, integration requires not only leaders who *proactively lead* integration, but also team members who *actively engage in* integration. If not, “the team runs the risk of not fully unpacking the integrative potential of a project or program” (Hoffmann et al., 2022a).

These recent studies notwithstanding, there are even fewer studies that combine existing conceptual and empirical insights to bridge the perceived gap between the theory and practice of ITD research (Zscheischler and Rogga, 2015; Huutoniemi et al., 2010) with a view to generating ‘how to’ practical knowledge (Fazey et al., 2018) on integration and integrative leadership (Deutsch et al., 2025a; Hoffmann et al., 2022b). Exceptions are studies that examine what conditions enable or hinder integration and integrative leadership at different structural levels (e.g., at individual, team, project, program, institutional and societal level), what challenges leaders face in terms of integration, what strategies they use to overcome such challenges, and what concrete actions different actor groups (e.g., individual team members, leaders, funders, directors, science-policy makers) can take to create more favourable conditions at different levels (Deutsch et al., 2025b). These studies identify core leadership challenges in terms of integration, including: a) mastering complexity and ambiguity, b) advancing decision-making with lateral leadership, c) ensuring responsibility and accountability, d) setting program boundaries, e) selecting suitable projects, and f) dealing with misconceptions. They also highlight respective strategies (structured according to attitudes, processes, and structures) to overcome such challenges and advance integration in ITD projects or programs (Deutsch et al., 2025a).

We argue that bridging this gap between the theory and practice of integration in ITD research to generate ‘how to’ practical knowledge on integration will support not only leaders and team members in *proactively leading* and *actively engaging in* integration, but also study directors and lecturers aiming at *effectively training* integration in ITD higher education. Although interest in such training is increasing, there are few studies that provide insights into how to design and implement integrative teaching and learning in ITD higher education (Vienni-Baptista and Hoffmann, 2024). Exceptions are studies that discuss teaching interdisciplinary integration at bachelor level (van Lambalgen and Van der Tuin, 2024) or graduate level (Eschen et al., 2025). Therefore, this study addresses two research questions: *i) How can we equip students, i.e. potential future leaders and team members of ITD projects or programs, with the key competencies necessary to lead and engage in integration across the boundaries of science, policy and practice? ii) What lessons learned can we derive from training integration in ITD higher education in order to support study directors and lecturers aiming at equipping students with the necessary competencies in integration?* We here refer to key competencies in integration - or integrative competencies as highlighted by van Goch (2023) or integrative ability as emphasized by (Eschen et al., 2025) - as complexes of knowledge, skills and attitudes (cf., Wiek et al. (2011)) that enable students to proactively lead and actively engage in integration.

We address these questions by giving a concrete account of our MSc course “Integration in Science, Policy and Practice: Inter- and Transdisciplinary Concepts, Methods, Tools” at ETH Zurich (Vienni-Baptista and Hoffmann, 2024) and by shedding light on *what* we teach and learn, *why and how*. We combine our conceptual insights into integration from studying integration (theory) and our empirical insights from leading and engaging in integration in different ITD projects and programs (practice) to first derive critical guiding principles for developing integrative teaching and learning in ITD higher education (cf., Bernert et al. (2022); Vienni-Baptista et al. (2024b)). Such principles guided us as co-lectures and co-authors in designing and implementing our MSc course on integration. We then describe our MSc course, outline key competencies and competence-oriented learning objectives, and illustrate how we applied the principles to design and implement different teaching and learning activities in our MSc course. We subsequently summarise learning outcomes as perceived by students based on students’ final presentations and course evaluations in order to draw lessons learned from training integration in our course. We conclude by outlining ways forward that can support current and future study directors and lecturers in their efforts to design and implement integrative teaching and learning in ITD higher education.

## Guiding principles for integrative teaching and learning

The flourishing of ITD research in the last 20 years (OECD, 2023) has had a gradually increasing influence on the question of how study directors and we - lecturers - can equip students with the key competencies necessary to design and implement ITD research in higher education. Since the 80’s, when the late Prof. Julie Thompson Klein mapped the many initiatives that had been developed in the US to foster ITD teaching and learning, many efforts have been institutionalised around the world with dissimilar outcomes (cf., Vienni Baptista and Klein (2022)). The common challenges of institutionalising ITD teaching and learning in higher education range from the lack of institutional flexibility (Vienni Baptista and Klein, 2022; Salmela et al., 2025) and the predominance of monodisciplinary structures (Lindvig, 2022; Vienni-Baptista et al., 2024b) to the difficulties of stakeholder engagement in teaching and learning (Pearce et al., 2018). Efforts to systematise best practices and lessons learned to train competencies in ITD research are on the rise. This particular issue provides one example of a larger community of practice being established among study directors, lecturers and students with a view to improving ITD teaching and learning in higher education against the backdrop of those shortcomings. Another example of such corrective efforts is the peer-to-peer exchange on innovative teaching and learning formats within the Global Alliance for Inter- and Transdisciplinarity (ITD Alliance) Working Group on Integration Experts and Expertise.

While ITD teaching and learning have been institutionalised around the world – though with dissimilar outcomes –, incorporating teaching and learning focused on integration remains a challenge for both study directors and lecturers (cf., Bernert et al. (2022)). Recent studies critically reflect on the concept of integration in teaching and learning (cf., van Lambalgen and Van der Tuin (2024); Szostak (2024); Vienni-Baptista and Hoffmann (2024)) with the seminal book *Interdisciplinary Research: Theory and Practice*, published by Repko (2008) and further editions with Szostak (2011, 2013, 2021), exploring, among other topics, the competencies needed to foster integrative teaching and learning in higher education (cf., Vienni-Baptista et al. (2024b)). The concept of integration has also been discussed in connection with developing sustainability programs in higher education (Brundiers et al., 2021), building expertise in transdisciplinary problem solving (Bammer et al., 2023), promoting the integrative ability of graduate students (Eschen et al., 2025) and rendering research societally relevant (Pohl et al., 2017; Vienni-Baptista et al., 2020) – to name a few examples.

**Fig. 1:**
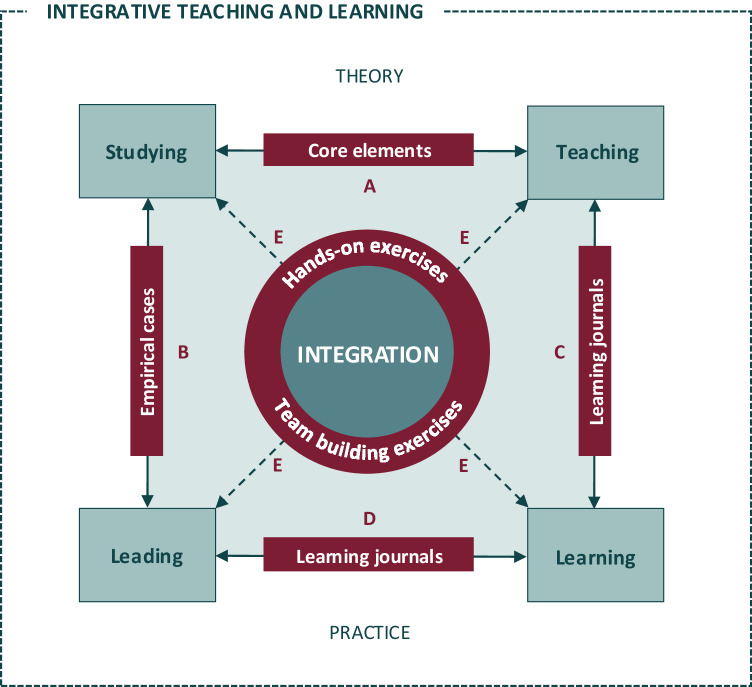
Integrative teaching and learning in higher education encompassing four cornerstones (green boxes): studying, leading, teaching and learning integration, and acknowledging the interplay between studying and teaching integration (arrow A), studying and leading integration (arrow B), teaching and learning integration (arrow C), learning and leading integration (arrow D) and studying, leading, teaching and learning integration (arrow E). Empirical cases, core elements of integration, hands-on and team building exercises, as well as learning journals (red boxes) substantiate such interplay between studying, leading, teaching and learning integration.

Building on recent calls to equip students with key competencies in integration (van Goch, 2023; Bammer et al., 2023; van Lambalgen and Van der Tuin, 2024; Eschen et al., 2025) and thus to train the next generation of leaders and team members (Roy et al., 2019), we link our conceptual insights from studying integration (theory)^1^ with our empirical insights from leading and engaging in integration as integration experts in different ITD projects or programs over the last 15 years (practice)^2^. By combing our conceptual and empirical insights, we derive guiding principles for designing and implementing integrative teaching and learning in higher education. We here define integrative teaching and learning as an iterative and reflexive process that includes “individual reflections on integration from different knowledge backgrounds and research cultures, collective discussions, and continuous adaptations of the course that lecturers perform when collaborating in a teaching and learning environment*”* (Vienni-Baptista and Hoffmann, 2024, p. 136). To our understanding, such integrative teaching and learning encompasses four cornerstones (green boxes, Fig. 1): studying, leading, teaching and learning of integration, and acknowledges the interplay between studying and teaching integration (arrow A, Fig. 1), studying and leading integration (arrow B, Fig. 1), teaching and learning integration (arrow C, Fig. 1) learning and leading (arrow D) as well as learning, leading, teaching and learning integration (arrow E). The guiding principles inform the interplay (arrows A-E, Fig. 1) between the four cornerstones (see Table 1). As further developed in section 3.1., empirical cases and the core elements of integration, hands-on and team building exercises as well as learning journals (red boxes, Fig. 1) allowed us, both lecturers and students, to substantiate such interplay and bridge conceptual and empirical insights into integration (left side, Fig. 1) with a view to teaching integration in theory and, concurrently, learning integration in practice (right side, Fig. 1).

**Table 1:** Guiding principles for integrative teaching and learning informing the interplay between studying, leading, teaching and learning integration in higher education.

Interactions	Guiding principles for integrative teaching and learning
Studying — Teaching (Arrow A)	• Identify core elements of integration (theories, concepts, strategies, dimensions, principles, roles, competencies) and draw on own experiences to illustrate core elements (bridging conceptual and empirical insights into integration) • Select mandatory readings on core elements and selected empirical cases to allow students to deepen their knowledge of integration • Integrate own critical reflections on integrative teaching and learning to serve students as a role model for integration and integrative leadership and encourage them to critically reflect on the course
Studying — Leading (Arrow B)	• Select empirical case studies (ITD projects or programs) suitable to illustrate core elements of integration and exemplify strategies, methods, and tools of integration • Invite experts involved in empirical cases to present the problem the ITD project or program addresses, outline the project’s or program’s integration strategy (including actor types, knowledge types as well as integration stages, methods, procedures) and detail one specific method or tool used within the project or program to integrate across science, policy and practice • Organise core elements as an integration rubric with keys and guides against which students analyse and compare integration across different empirical cases in a structured and systematised way
Teaching — Learning (Arrow C)	• Use teaching and learning journals as a prompt for individual work in which students and teachers critically reflect on course modules • Use integration rubrics as prompts for group work in which students design, plan and implement their own integration processes (i.e., analyzing and comparing empirical cases in terms of integration) and generate their own integrated outputs (consolidated analysis and cross-comparison) • Design hands-on exercises to enable students to apply and experience integration methods or tools used within case studies and assess their strengths and weaknesses in terms of integration
Learning — Leading (Arrow D)	• Use group work as a means for experiential learning to enable students to draw on their direct experiences in proactively leading and actively engaging in their own integration process and critically reflect, think and act on the various challenges and opportunities they experience in their collaborative and integrative effort • Use teaching and learning journals as a prompt for individual work to allow students and teachers to critically reflect on group work (students) and integrative teaching and learning (lecturers)
Studying — Learning — Leading — Teaching (Arrow E)	• Dedicate time to team building exercises to create an experimental space as well as a safe, positive and constructive environment in which students are motivated to contribute and draw on one another’s contributions, in which students feel effectively heard, appropriately considered and adequately credited for their contributions and in which students can apply, experience and experiment with different integration methods and tools

The four cornerstones of integrative teaching and learning and their interplay, as illustrated in Fig. 1, can be seen from a lecturer’s perspective, i.e. lecturers from different knowledge backgrounds and research cultures – as in our case – combining their individual insights from leading and studying integration in different ITD projects or programs (empirical cases) with a view to teaching students integration (core elements) and thereby learning how to integrate not only their different perspectives on integration, but also their different theories, concepts, methods and tools of integration (learning journals) (Vienni-Baptista and Hoffmann, 2024). They can also be viewed from a student’s perspective, i.e. students from different knowledge backgrounds and research cultures drawing on conceptual and empirical insights gained from ITD projects or programs (empirical cases) to study the core elements of integration; they lead their own interdisciplinary integration process and thereby learn how to integrate their perspectives on integration by applying the core elements to empirical cases; by critically reflecting on the course and their own integration process (learning journals) and sharing their insights throughout the course and in their final presentation, they also teach lecturers potential ways forward in terms of strengthening integrative teaching and learning.

## An example of integrative teaching and learning

The MSc course “Integration in Science, Policy and Practice: Inter- and Transdisciplinary Concepts, Methods, Tools” offered in the environmental sciences program at ETH Zurich has been developed in an iterative and reflexive process (Vienni-Baptista and Hoffmann, 2024). The process included multiple rounds of bilateral discussions between us, as the co-lecturers, about (a) *what* conceptual and empirical insights from studying and leading integration in ITD research projects or programs can feed into teaching and learning on integration, (b) *how* to translate these insights into key competencies and competence-oriented learning objectives and c) *how* to design teaching and learning activities to ensure constructive alignment (Biggs and Tang, 2011) with such competence-oriented learning objectives. Once we had designed our teaching and learning activities, we implemented these activities in a first edition of the course in the spring semester of 2023 (13 weeks à 2 hours) with a cohort of 13 students mainly from the Department of Environmental Sciences (D-USYS), but also from the Department of Civil, Environmental and Geomatic Engineering (D-BAUG) (2 students) and the Humanities, Social and Political Sciences (D-GESS) (1 student). We revised the course and offered it again in the spring semester of 2024 and 2025 (13 weeks à 2 hours) with a cohort of 17 students (2024) and 19 students (2025) mainly from D-USYS and D-BAUG (1 student).

**Fig. 2:**
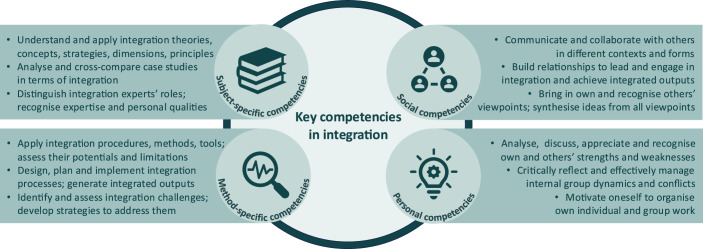
Four types of key competencies, i.e. subject- and method-specific competencies, social and personal competencies, to proactively lead and actively engage in integration and their operationalization through competence-oriented learning objectives of the MSc course “Integration in Science, Policy and Practice: Inter- and Transdisciplinary Concepts, Methods, Tools” at ETH Zurich, based on La Cara (2022).

We built on the ETH Zurich Competence Framework, which is a compilation of twenty competencies that our university fosters in order to train students in the knowledge, skills and attitudes of high societal value (La Cara, 2022), and selected four types of key competencies to proactively lead and actively engage in integration, namely subject- and method-specific competencies as well as social and personal competencies (Vienni-Baptista and Hoffmann, 2024). We operationalised these key competencies through competence-oriented learning objectives, as illustrated in Fig. 2 and summarised in Table 2, by drawing on our insights from studying and leading integration.

**Table 2:** Key competencies in integration and their operationalization through competence-oriented learning objectives of the MSc course “Integration in Science, Policy and Practice: Inter- and Transdisciplinary Concepts, Methods, Tools” offered in the environmental sciences program at ETH Zurich based on La Cara (2022).

Key competencies	Competence-oriented learning objectives
Subject-specific competencies	Students are able to: (i) understand theories, concepts, strategies, dimensions and principles of integration and apply these to selected case studies; (ii) analyse and cross-compare selected case studies in terms of integration, break down integration into parts and understand their interactions; (iii) distinguish integration experts’ diverse roles and recognise required expertise and personal qualities.
Method-specific competencies	Students are able to: (i) apply different integration procedures, methods and tools in diverse contexts and assess their potentials and limitations; (ii) design, plan and implement integration processes and generate integrated outputs; (iii) identify and assess challenges inherent to integration; and (iv) develop strategies to address them.
Social competencies	Students are able to: (i) communicate and collaborate with others in different contexts and forms; (ii) build relationships with others to lead and engage in integration processes and achieve integrated outputs; (iii) bring in their own viewpoints and recognise other viewpoints; and (iv) synthesise ideas from all viewpoints in a constructive and creative manner.
Personal competencies	Students are able to: (i) analyse and discuss own strengths and weaknesses and recognise and appreciate strengths and weaknesses of others; (ii) critically reflect and effectively manage internal group dynamics and conflicts; and (iii) motivate themselves to organise own individual and group work in order to achieve integrated outputs.

## Teaching and learning activities

The following section illustrates how our guiding principles (section 2) can be applied to develop integrative teaching and learning activities. It outlines: a) core elements of integration, b) empirical cases, c) hands-on and team building exercises, and d) learning journals that enabled us as co-lecturers to bridge between our conceptual and empirical insights from studying and leading integration (left side, Fig. 1) with a view to teaching students integration in theory (e.g., understanding, analysing, distinguishing, combining, discussing, reflecting and assessing integration) and, concurrently, enabling them to learn integration in practice (e.g., applying, experiencing and experimenting with integration methods and tools; designing, planning and implementing integration processes; generating integrated outputs) (right side, Fig. 1).

### Core elements

We designed the course to address the core elements of integration in the first four weeks (Vienni-Baptista and Hoffmann, 2024):

- *Theories* and *concepts* of integration, including different understandings of integration in disciplinary, multi-, inter- and transdisciplinary research (Tress et al., 2005; Vienni-Baptista et al., 2022), different features of integration (O’Rourke et al., 2016; Pohl et al., 2021), different purposes of integration (Hoffmann et al., 2017a; Hoffmann et al., 2022b), different stages of ideal typical ITD research processes (Pohl et al., 2017; Hoffmann et al., 2019) and ideal typical policy processes (Wuelser et al., 2012) as well as different enabling conditions for integration (Deutsch et al. 2025);
- *Strategies* of integration, including different stages, actors, procedures, methods and knowledge types and their constructive combination (Rossini and Porter, 1979; Bergmann et al., 2012; CASS and ProClim, 1997; Hoffmann, 2016; Hoffmann et al., 2017b);
- *Dimensions* of integration, including cognitive, social, emotional, spatial, temporal and strategic dimensions (Pohl et al., 2021; Freeth and Caniglia, 2019), and different *principles* of integration, including context-based, pluralistic, goal-oriented, interactive (Norström et al., 2020; Fazey et al., 2018; Briers and Vienni Baptista, under review);
- *Role*s of researchers in ITD projects or programs (Bulten et al., 2021; Hofmann et al., 2025), including the diverse roles of *integration experts*, their *personal qualities* and *expertise* in integration (Hoffmann et al., 2022a; Hoffmann et al., 2024).

To link conceptual insights on integration *(studying integration)* with empirical insights *(leading integration)*, we drew on our experiential knowledge in leading and engaging in integration in different ITD projects and programs and used these projects and programs as concrete examples to exemplify the core elements listed above to: (a) illustrate theories, concepts, strategies, dimensions and principles *(subject-specific competencies)*, (b) elucidate integrative procedures, methods and tools *(method-specific competencies)*, and (c) discuss integration experts’ diverse roles, personal qualities and expertise *(subject-specific competencies)* (Vienni-Baptista and Hoffmann, 2024). In addition, we selected mandatory readings and additional resources to allow students to deepen their knowledge of core elements and empirical cases *(subject-specific competencies)* (Pohl et al., 2021; Bulten et al., 2021; Fazey et al., 2018; O’Rourke, 2017; Hoffmann et al., 2022a; Vienni-Baptista et al., 2022) and developed a learning journal as a prompt for individual work (Vienni-Baptista and Hoffmann, 2024).

### Empirical cases

Based on this introduction, in the next eight weeks four selected empirical case studies (ITD projects or programs) are presented, with one case study being discussed every second week. “During the first session [week 5, 7, 9, and 11], [invited] experts provide an overview of the case, touching upon the [core] elements presented in the first four weeks of the course and highlighting (i) the particular environmental or societal problem addressed, (ii) the integration process and related outputs pursued, and (iii) the integration methods and tools used to combine perspectives from different disciplines, as well as from science, policy and practice” (Vienni-Baptista and Hoffmann, 2024, p. 140).

To analyse and compare inter- and transdisciplinary integration across selected empirical case studies in a structured and systematised way, we developed an integration rubric with keys and guides on the various core elements introduced in the first four weeks. We use this rubric as a prompt for group work in which students engage with the four case studies and jointly analyse and cross-compare the cases in groups of 3-4 students in terms of integration; i.e. students apply the conceptual and empirical insights gained during the first four weeks to the four selected cases and derive key insights into the various core elements for each individual case study *(subject-specific competencies)*; they then cross-compare the different cases in terms of integration, discuss similarities, differences and complementarities and synthesise key insights from their cross-comparison *(subject-specific and social competencies)*. The final consolidated analysis and cross-comparison (mandatory assignment) is handed over to the lecturers at the end of the course.

We use this group work as a means for students’ experiential learning. We refer to experiential learning as “a cyclical process of concrete learning, reflective observation, abstract conceptualization and active experimentation*”* (Lotrecchiano and Misra, 2020, p. 39) on how to design, plan and implement their own interdisciplinary integration processes (i.e., analyzing and comparing empirical cases in terms of inter- and transdisciplinary integration) and generate their own integrated outputs (consolidated analysis and cross-comparison of empirical cases) *(method-specific competencies)*; i.e., students draw on their direct experiences (Freeth and Caniglia, 2019) in proactively leading and actively engaging in the joint analysis and cross-comparison of empirical cases. They use the keys and guides provided by the rubric and critically reflect, think and act (Kolb et al., 2014) on the various challenges and opportunities they experience not only in leading and engaging in this collaborative and integrative process, but also in achieving a final integrated output *(social competencies)* (Hoffmann et al., 2022b). As students have only limited time to analyse and cross-compare all four cases in the classroom, they need to self-organise and continue their group work outside the classroom to complete their mandatory assignment *(personal competencies)*. As part of this assignment, we ask students to prepare a final joint group presentation, including three components: (i) individual insights into integration from students’ learning journals, (ii) consolidated insights into internal group dynamics from students’ learning journals and joint group reflections, and (iii) key findings from the completed group rubric. Students are free to choose their own format and develop their own idea on how to structure the final presentation on condition that all members actively engage in sharing their insights and findings on all three components.

### Hands-on and team building exercises

“During the second session [week 6, 8, 10, and 12], the same [invited] experts apply—together with the students and main lecturers—the integration method and tools used in the case study. Applying such methods [and tools] allows students to embody some of the challenges and opportunities” (Vienni-Baptista and Hoffmann, 2024, p. 140) integration implies *(method-specific competencies)*. For example, students develop in smaller groups in session 12 Theories of Change (ToC) based on the question “What could a future look like at ETH Zurich in which inter- and transdisciplinary integration has been strengthened in higher education”. Building on the conceptual and empirical insights into integration gained throughout the course, students engage in and build on their own perspectives to first define a visionary, but realistic impact statement, and then - starting from the desired impact and moving backwards - identify necessary long-, medium- and short-term changes (‘backcasting’) that need to be in place to achieve the desired impact (Deutsch et al., 2021). They then identify potential interventions, i.e. a set of deliberate activities that students can implement in collaboration with other actors such as study directors, lecturers, professors, student associations, etc., that aim at contributing to change in higher education. Engaging in and building on their different perspectives while developing their own ToC enables students to embrace the messiness of interdisciplinary integration in the classroom and experience the challenges and opportunities of integrating their very different perspectives on how and why change is expected to happen at ETH Zurich. It also allows them to experiment with a particular integration method or tool (here: ToCs) and to assess their own strengths and weaknesses, potentials and limitations in terms of integration.

We have developed manuals and questions to guide students on how to both thoroughly apply the various integration methods and tools as well as to subsequently critically reflect on the integration process and the integrated output that emerges from the process. Such manuals and questions included keys and prompts on the what, why, when, how, and so what of such methods and tools in the integration rubric *(method-specific competencies)*. Applying different integration methods and tools in the classroom, but also jointly analysing and cross-comparing empirical case studies, allow students to assume different roles of an integration expert and critically reflect on the challenges and opportunities of these roles as well as the different kinds of knowledge, skills and attitudes required to play such roles (Hoffmann et al., 2024) *(subject-specific competencies)*.

To create an experimental and safe space in which students are able to apply, experience and experiment with different integration methods and tools, we dedicate from the outset explicit time for team building exercises (cf., Bernert et al. (2022). Such exercises include, for example, playing bingo (Schrader, 2022) to get to know each other, which incorporate questions on students’ personal background, societal engagement or academic trajectory. Exercises also involve developing team charters (Edelbroek et al., 2018) to share own strengths and weaknesses and recognise and appreciate the strengths and weaknesses of others. Such team building exercises help to create a positive environment in which students are committed to listen to each other and learn from each other (Mezirow, 1997; Bammer et al., 2020) and are motivated to contribute their own perspectives, recognise different perspectives and draw on each other’s perspectives in a constructive and creative manner (Harvey et al., 2018) *(social and personal competencies)*.

### Teaching and learning journals

We use teaching and learning journals as a prompt for individual work (mandatory assignment). During the initial version of the course, we realised that the questions that guided students and lecturers in their reflections on the course content, group dynamics and students’ competencies were too exhaustive. For the second and third versions, we have restructured and simplified our teaching and learning journal for both students and lecturers, building on questions linked to experiential learning theory (*what* happened*, why, so what* and *now what*) (Kolb et al., 2014). In our course, we ask students to individually reflect on these questions after each course module or group work and bring their individual reflections to the classroom to share key insights within their group (Vienni-Baptista and Hoffmann, 2024). The shared and consolidated key insights from their individual learning journals and joint group reflections form part of the final group presentation *(social and personal competencies)*. For more details regarding the use of teaching and learning journals as an integrative tool, see Vienni-Baptista and Hoffmann (2024).

## Learning outcomes and lessons learned

In this section, we discuss the learning outcomes of our course – defined as an observed state of what students are able to do (Brundiers et al., 2021) – as perceived by our first cohort of students and derive lessons learned by us co-lecturers. Given that reflexivity is a central component of our integrative teaching and learning, we created several moments to discuss such learning outcomes not only with students, but also with peer colleagues, educational developers and between ourselves.^3^ Additionally, we organised a seminar with peer colleagues and educational developers to present our lessons learned and discuss the key competences, competence-oriented learning objectives and learning outcomes as perceived by our first cohort of students. We invited students who had attended the initial version of our course to participate in the seminar and give voice to their perspectives. Together with the final group presentations of the first cohort of students, which included a critical reflection on our course, these different moments allowed us to assess the learning outcomes of our course as perceived by our students.^4^

To identify the lessons learned, we qualitatively clustered similar learning outcomes in groups and related them to the corresponding key competencies and competence-based learning objectives summarized in Table 2. After completion of our empirical analysis, we invited students to review an earlier version of this section on learning outcomes and lessons learned and to validate (or refute) our interpretations. In the following we summarize learning outcomes as perceived by our first cohort of students based on their group reflections and summarise some lessons learned by us co-authors and co-lecturers based on such reflections.

### Learning outcome 1: Understand and apply concepts of integration

Organising core elements of integration in a rubric with keys and guides to analyse and cross-compare empirical cases allowed students to deepen their understanding of different theories and concepts of integration. Although they consistently described the mandatory readings as complex and “difficult-to-grasp”, they acknowledged the usefulness of empirical cases to exemplify such core elements and bridge between the theory and practice of integration. They also recognised the usefulness of learning journals and group work to individually and jointly reflect on key insights into integration. During the final group presentations, students indicated:

*“The fact that we were discussing together [in our group], […] it really helped us build a common language and actually understanding the different concepts we read in the research papers. […] We discovered during the case [comparison] that integration is extremely case dependent. […] When we were reading some papers, like the fundamentals of integration, I don’t think it was appropriate because [integration] is super different for each project. But at the same time, those papers add some insights that are really useful for PIs that are trying to do integration in the project.” (Group 3, Oral Presentation 2024)*.

The students’ critical reflections on our mandatory readings offer some important lessons learned on the ITD literature, which involves ideal-typical conceptualisations that are often abstract and difficult to understand and apply to real contexts. Developing tailored educational resources to teach, learn, lead and study ITD in general and integration in ITD projects or programs in particular (as, for example, Vienni-Baptista et al. (2024b)) might offer a way forward. Such mandatory readings may be well accompanied by learning journals as pedagogical devices to connect the theory and practice of integration and draw lessons learned, as exemplified in the following quotes:

*“This is not a complete list, but these themes came up again and again in all of our learning journals. First of all, integration creates a space to exchange knowledge and secondly, it creates co-ownership. We came to the conclusion that [co-ownership] is very powerful because it creates […] a feeling of control, of influence, and a sense of responsibility for the output, which then enhances the acceptance of outcomes from all participants*.” *(Group 3, Oral Presentation 2024).*

The complex and ‘difficult-to-grasp’ literature in the field of ITD research poses a challenge for integrative teaching and learning, namely to bridge the divide between the theory and practice of integration and to substantiate high-level conceptualisations with empirical examples. Ideally, lecturers embody such examples, provide insights into their own struggles on the way towards integration and share their own experiences on ‘how to’ lead and engage in integration in different ITD projects or programs. The following quote gives an example of such ‘how to’ practical knowledge:

*“The first thing as we learned through our group work is to create an atmosphere in which everybody feels comfortable and to build personal relationships and get to know each other. This might look or might seem in the beginning that it’s not really efficient […]. But in the end, because you know each other better […] that actually leads to a better output. In the end, it is actually more efficient than if you just focus on the short-term efficiency of getting work done.” (Group 3, Oral Presentation 2024)*.

The students’ critical reflections illustrate their ability to grasp the core elements of integration at a conceptual level and acknowledge the variety of integration processes and related outputs across different case studies (theory). They also manifest their ability to apply these core elements and discuss, reflect and cross-compare selected cases in terms of integration by using the integration rubric with its keys and guides as prompts (practice) *(subject-specific competencies)*. They further highlight their ability to bring in their different insights (collected in their mandatory learning journals), analyse them and identify overarching patterns as part of their mandatory group work, and to synthesise them in terms of key requirements for integration *(subject-specific and social competencies)*. The following quote is indicative:

*“By being experimental, you can really increase your learning rate because also when we were trying, for example, to work on the rubric or on the concepts, in the beginning we just learned about the theory and we found it really hard to actually apply it. But then, by comparing the cases, we felt like we learned a lot more because we were actually applying it.” (Group presentation 3, Oral presentation 2024)*.

### Learning outcome 2: Apply and assess different methods and tools of integration

Designing hands-on exercises in the classroom allowed students to lead and engage in their own interdisciplinary integration process and to reflect and act on the challenges they encountered on their way towards integration, for example, in their mandatory group work. Such hands-on exercises enabled them to experiment with and apply different methods and tools of integration and to assess their potentials and limitations in, for example, navigating power dynamics in teams and projects. Students stated in their group presentation:

*“What we found was that through the integration methods that we learned about, talked about and tried out, we found that there’s not really one right way how to organise your team or your project. There are many trade-offs, advantages and disadvantages to every single case. We found it interesting that this highly influences on which […] integration procedure [and method] you want to use. Or the other way around: if you may favour a specific procedure [and method], then you also need to organise your team and your project differently.” (Group presentation 3, Oral Presentation 2024).*

The students’ critical reflections underline their ability to assess different integration procedures and methods in terms of their strengths and weaknesses *(method-specific competencies)*; they also illustrate their ability to lead their own integration process, to critically reflect on it and to act on the challenges they experienced by adapting their process from, for example, first dividing the tasks within the group to then organising reporting sessions to subsequently sharing key insights gained from their task division to, finally, coming up with an integrated output *(social competencies)*. The following quote is indicative:

*“Early on in the course we agreed to divide our tasks and then work individually. […] Each of us were working individually and we quickly realised that it might be better to increase the interaction between the four of us. So, we decided to have reporting sessions and discussions between us and talk what we focus on […] and learn in individual parts, and explain that to other people in the group.” (Group presentation 3, Oral Presentation 2024)*.

Time in the classroom is limited. It undoubtedly means that ‘less is more’ and requires focusing on selected key methods and tools that are first introduced by lecturers and then applied by students, allowing them to bridge between the theory and practice of integration, to lead their own integration process as a means for experiential learning (Kolb et al., 2014), and to test different ways of working within the group, as these quotes show:

*“I was in charge of several questions in the rubric and I would explain what I learned and tell them to others. This way we could increase the learning amount in our group. Later on, especially after starting the preparation for this presentation, we really started to integrate our own individual insights as well as our group insights, we could enhance our integration*.

*For us, the word ‘distribution’ [of tasks] worked pretty well. In addition to that, the discussion and reporting sessions were really useful to share our insights. We felt that we could have an open communication, transparency, and also reliability that everyone would do their own individual part for the rubric. This way, we could feel comfortable working in this group together.” (Group presentation 2, Oral Presentation 2024)*.

The students’ critical reflections offer some lessons learned for integrative teaching and learning, namely to consider the classroom as an ‘experimental’ space that allows students to explore different methods and tools – and to ‘play’ with them in a protected niche (Vienni-Baptista and Hoffmann, 2024). Building such experimental space into integrative teaching and learning means shifting priorities from ‘purely’ teaching integration in theory (concepts, methods, tools) to ‘actively’ learning integration in practice by applying concepts, methods and tools in diverse contexts. It thus means balancing the theory and practice of integration in the classroom (cf. Fig. 1).

### Learning outcome 3: Reflect on own roles and competencies in integration

By allowing students to proactively lead and actively engage in integration in the classroom and by discussing researchers’ diverse roles in ITD projects and programs, we enabled students to critically reflect on their competencies in integration throughout the course (see teaching and learning journals). It allowed them to act on their strengths and weaknesses before embarking on the mandatory group work, as highlighted in one group presentation:

*“It started with a good discussion at the beginning of the semester where we each discussed how we work personally, what works best for us, what our strengths and weaknesses are, what my groupmates’ strengths and weaknesses are, and then we agreed how we want to work as a group.” (Group presentation 1, Oral Presentation 2024)*.

Students’ critical reflections illustrate their ability to distinguish the different roles students assumed in their group work and to recognise and appreciate their own strengths and weaknesses as well as the those of others related to such roles *(personal competencies)*. They also show their ability to critically reflect, think and act upon the challenges they experienced in the group work and to effectively manage group dynamics to ensure a final integrated output *(social competencies)*. They also show their ability to communicate and collaborate with each other, to build relationships, to bring in their own perspectives and to negotiate and reconcile different perspectives, as the following quotes emphasise:

*“It was really interesting to work together because we felt [person xxx] is a very creative and open-minded thinker who often thinks out of the box and has very creative ideas. S/he is very dedicated to create an atmosphere in which everybody felt comfortable and that we all had fun doing our work. So, we appointed him/her to be our Chief Happiness Officer in our group […]. That was really helpful*.

*On the other side, we have [person yyy] who is a very critical thinker in many different areas because s/he always brought critical perspectives about the transdisciplinary and interdisciplinary integration methods. But they also reflected on whatever ideas we brought up […]. Somehow, it was a bit hard to bring all these points and different characteristics together—I was able to sometimes keep the big picture in mind and also negotiate between these different perspectives and opinions. We also try to assign a bit the different roles that we learned about in the course.” (Group presentation 3, Oral Presentation 2024)*.

The students’ critical reflections provide some lessons learned on the need to dedicate sufficient time and space from the beginning to team building exercises to create a positive and constructive environment in which students are open to share their own strengths and weaknesses and to talk and collaborate with each other – also with a view to achieving an integrated output. The following quote is indicative:

*“I think a lot of different research programs and projects have the potential to be integrative, but it’s really how the researchers and the people involved in the project are intentional in actually fostering and facilitating integration within their roles that they play, as well as being adaptive to assume and take on other roles as we’ve heard […]. I think it was very helpful in this class to actually see the different ways in which integration manifests in research and academia and hopefully, eventually apply to policy.” (Group presentation 4, Oral Presentation 2024)*.

Lecturers can nourish a safe space by discussing their own strengths and weaknesses as collaborators in teams and co-lecturers in the classroom and/or by sharing their own reflections on the challenges in studying, leading, teaching and learning integration and the strategies they employ to overcome them. They can thus act as role models in creating a positive and constructive environment so that students’ collaborative and integrative efforts thrive in the classroom and beyond.

## Conclusion

Our study set out to explore the question: *i) How can we equip students, i.e. potential future leaders and team members of ITD projects or programs, with the key competencies necessary to lead and engage in integration across the boundaries of science, policy and practice?*

We addressed this question by providing empirical insights into our MSc Course “Integration in Science, Policy and Practice: Inter- and Transdisciplinary Concepts, Methods, Tools” at ETH Zurich in Switzerland. We derived guiding principles for integrative teaching and learning, defined key competencies in integration, operationalized such competencies through competence-oriented learning objectives and outlined our teaching and learning activities. Giving such concrete account of *what* we teach and learn, *why* and *how*, we aim to support current and future study directors and lecturers in their efforts to design and implement integrative teaching and learning in ITD higher education.

To conclude, we come back to the second question guiding this study: *ii) What lessons learned can we derive from training integration in ITD higher education to support study directors and lecturers aiming at equipping students with the necessary competencies in integration?*

Based on our experience, we identified three main lessons from designing and implementing integrative teaching and learning in ITD higher education. First, “*embedded learning*” provides a solid ground for training integration in the classroom. In such classroom-based learning, students and lecturers play a crucial role in actively engaging in sharing both conceptual and empirical insights into integration as well as the challenges they – students and lecturers – faced in leading, studying, teaching and learning integration, including the strategies adopted to surmount them. For example, we co-lecturers openly discussed the competence-based learning objectives with our students with a view to explicating our aim to equip students with the key competencies necessary to proactively lead and actively engage in integration, but also to trigger critical reflections on the learning outcomes as perceived by our students (see previous section). This allowed us, co-lecturers, to re-think such key competencies and competency-based learning objectives and – perhaps more importantly – to redesign the interplay between subject- and method-specific as well as social and personal competencies with a view to achieving integrative teaching and learning in the classroom.

Second, the strong connection between studying, leading, teaching and learning integration (Fig. 1) as well as the diverse empirical case studies – informed by experts’, lecturers’ and students’ insights into integration as both leaders and team members in ITD projects or programs – allows for “*nuanced learning*” in the classroom. Here again, co-lecturers and experts play a crucial role in coaching, supporting and accompanying students in their own learning journey and in clarifying the relevance of the underlying guiding principles of their integrative teaching and learning, thereby promoting students’ critical reflections on integration at individual and collective level. In this way, for example, students were empowered to diversely experiment with and experience their own interdisciplinary integration processes in the classroom (and beyond), while joint discussions and reflections among students, co-lecturers and experts furthered common awareness of the nuanced experiences with these processes.

Third, *“constructive alignment”* between the three components – the intended curriculum (objectives and outcomes), the enacted curriculum (teaching and learning activities) and the experienced curriculum (students’ perceptions) (cf., Hounsell and Hounsell (2007)) – requires a continuous revision of conceptual and empirical insights taught in the first four weeks of our course. Such revision is a prerequisite for integrative teaching and learning (Vienni-Baptista and Hoffmann, 2024) as the ITD field is constantly evolving and new conceptual and empirical insights, particularly into integrative leadership (Deutsch et al., 2025a), enabling conditions for ITD integration (Deutsch et al., 2025b; Vienni-Baptista et al., 2024a) and interdisciplinary practices in higher education (Vienni-Baptista et al., 2024b), are being generated. This poses a challenge not only in terms of how we co-lecturers teach integration concepts, methods and tools in theory, but also how students learn to apply such concepts, methods and tools in practice. Drawing on the four cornerstones of integrative teaching and learning, i.e. studying, leading, teaching and learning integration and the guiding principles that inform their interplay, constitutes an important step towards bridging the perceived gap between the theory and practice of integration in ITD research and ITD higher education.

Our study fills part of this gap by providing ‘how to’ practical knowledge on how to design and implement integrative teaching and learning in ITD higher education. The results of our study are embedded in the specific context of a particular MSc course at ETH Zurich in Switzerland. We consider our results relevant for study directors and lecturers at other universities aiming at equipping students, potential future leaders and team members of ITD projects or programs, with the necessary key competencies in integration. One line of future research for us co-lecturers and co-authors is to develop assessment tools for evaluating the extent to which students develop key competencies in a more structured manner. Applying our guiding principles to online MSc courses would provide further insights into the four cornerstones of integrative teaching and learning and their interplay in ITD higher education.

## Data Availability

The data analyzed during the current study are not publicly available but are available from the corresponding author on reasonable request.

## Footnotes

1 Hoffmann (2016); Hoffmann et al. (2017b, 2017a); Hoffmann et al. (2022a); Hoffmann et al. (2022b); Deutsch et al. (2021); Deutsch et al. (2025a); Deutsch et al. (2025b); Pohl et al. (2021); Vienni-Baptista et al. (2024a).

2 National Research Program “Sustainable Water Management” (NRP 61), Eawag Discretionary Fund Project “Water and sanitation innovations for non-grid solutions” (Wings), Sinergia Project “Transformation in Pesticide Governance” (TRAPEGO), Sinergia Project “Transformation toward resilient ecosystems” (TREBRIDGE), EU Horizon Europe Project “Delivering the next generation of open integrated assessment models” (DIAMOND); Swiss Federal Office of Energy Project “Co-Evolution and Coordinated Simulation of the Swiss Energy System and Swiss Society” (CoSi) and PRIMA Grant “Investigating interdisciplinarity and transdisciplinarity: intersections among practices, culture(s) and policy” (Intersections).

3 ETH Zurich sets up annual teaching evaluations in which students can identify key aspects they would suggest changing or maintaining.

4 To be able to substantiate such learning outcomes, we asked the first cohort of students for permission to audio record their final group presentations, transcribed the audio recording and encoded these group presentations. We applied the qualitative methodology of grounded theory (Glaser and Strauss, 1967) to derive from these final group presentations of the first cohort key learning outcomes as perceived by our students. Informed consent was obtained from the first cohort of students (25 ETHICS-057, February 25, 2025, ETH Zurich).
